# Power or speed: Which metric is more accurate for modelling endurance running performance on track?

**DOI:** 10.1002/ejsc.12210

**Published:** 2024-10-14

**Authors:** Santiago A. Ruiz‐Alias, Alberto A. Ñancupil‐Andrade, Alejandro Pérez‐Castilla, Felipe García‐Pinillos

**Affiliations:** ^1^ Department of Physical Education and Sports Faculty of Sport Sciences University of Granada Granada Spain; ^2^ Sport and Health University Research Center (iMUDS) University of Granada Granada Spain; ^3^ Department of Health Los Lagos University Puerto Montt Chile; ^4^ Department of Education Faculty of Education Sciences University of Almería Almería Spain; ^5^ SPORT Research Group (CTS‐1024) CIBIS (Centro de Investigación para el Bienestar y la Inclusión Social) Research Center University of Almería Almería Spain; ^6^ Department of Physical Education Sports and Recreation. Universidad de La Frontera Temuco Chile

**Keywords:** endurance performance, modelling, running

## Abstract

This study aimed to compare the accuracy of the power output, measured by a power meter, with respect to the speed, measured by an inertial measurement unit (IMU) and a global navigation satellite system (GNSS) sport watch to determine the critical power (CP) and speed (CS), work over CP (W') and CS (D'), and long‐duration performance (i.e., 60 min). Fifteen highly trained athletes randomly performed seven time trials on a 400 m track. The CP/CS and W'/D' were defined through the inverse of time model using the 3, 4, 5, 10, and 20 min trials. The 60 min performance was estimated through the power law model using the 1, 3, and 10 min trials and compared with the actual performance. A lower standard error of the estimate was obtained when using the power meter (CP: 2.7 [2.1–3.3] % and W': 13.8 [10.4–17.3] %) compared to the speed reported by the IMU (CS: 3.4 [2.5–4.3] %) and D': 20.7 [16.6–24.7] %) and GNSS sport watch (CS: 3.4 [2.5–4.3] % and D': 20.6 [16.7–24.7] %). A lower coefficient of variation was also observed for the power meter (4.9 [3.7–6.1] %) Regarding the speed reported by the IMU (10.9 [7.1–14.8] %) and GNSS sport watch (10.9 [7.0–14.7] %) in the 60 min performance estimation, the power meter offered lower errors than the IMU and GNSS sport watch for modelling endurance performance on the track.

## Highlights

- Recent advances in wearable technology are creating new opportunities to accurately monitor running intensity in contexts where precision is essential (i.e., track running) for athletes' (e.g., pacing) and coaches' (e.g., performance analysis) tasks.
- The track mode of recent global navigation satellite system (GNSS) sport watches seems to offer a decent improvement. Alternatively, new inertial measurement units (IMUs) designed specifically for running have emerged offering the capability to monitor distance in specific contexts such as treadmills and indoor tracks. Additionally, these IMUs provide new metrics of the external load.
- The power meter provided by the Stryd inertial measurement unit (IMU) demonstrated lower estimation errors in modelling endurance running performance on the track compared to speed data reported by the same IMU and a Garmin GNSS sports watch (using the track mode).

## 1 INTRODUCTION

Current advances in wearable technology are opening the chance to new forms of running monitoring. From the inception of sport watches using the GNSS, tracking speed was the unique measure of the external load. However, the power metric in running arose as a potential alternative for specific contexts. Derived from undisclosed equations, the power metric is nowadays reported by different IMUs, being sensitive to different external load factors such as speed, slope, and body mass (Cerezuela‐Espejo et al., 2020). Specifically, from the different commercial IMUs available, the Stryd power meter has demonstrated strong reliability and level of agreement with the external work and oxygen uptake in different environments (i.e., indoor and outdoor) and conditions (i.e., speed, body weight, and slope), being thus the wearable of reference in this area (Cerezuela‐Espejo et al., 2021; García‐Pinillos et al., 2019; Imbach et al., 2020; Ruiz‐Alias et al., 2022; Taboga et al., 2022).

Accordingly, different studies have addressed the question of which metric, the speed or power output, is more suitable to monitor in different running contexts (Hingrand et al., 2023; Van Rassel et al., 2022, 2023). On the one hand, in the study of Van Rassel et al. (Van Rassel et al., 2023), athletes completed different distances to determine the critical speed (CS) and critical power (CP) on an outdoor track. Then, athletes performed a single 800 m trial at CS, observing that the mean power output did not differ from the CP, being therefore interchangeable. On the other hand, Van Rassel et al. (Van Rassel et al., 2022) tested the validity of the speed and power output metrics to illustrate the internal load (i.e., oxygen uptake) associated to the maximal metabolic steady state using a specific treadmill protocol composed of longer steps (i.e., 12 min) than the 800 m trial. In this case, only the power output was able to capture the actual internal load, being underestimated when using the speed metric, probably due to the misalignments between the speed and the slow component of the oxygen uptake. In this regard, it is worth noting that the slow component has been accompanied by increased potential work (Borrani et al., 2003), a variable captured by the Stryd power meter (Cerezuela‐Espejo et al., 2020; Imbach et al., 2020). Lastly, Hingrand et al. (2023) reinforced the role of the power metric to better illustrate the internal load than speed on ascension. The subjects completed two identical graded exercise tests in terms of ascensional speed increments, differing only in their slope (i.e., 10 vs. 25%) (i.e., athletes were ascending identical meters but at different speeds). The results revealed that the power output was able to illustrate the internal load of each ascensional speed irrespective of the slope, but not the ascensional speed itself, reflecting a superior internal load at 10% compared to the 25% slope.

In addition to these contexts, the comparison of power and speed metrics is also crucial in other training and testing scenarios. On the one hand, the CP or CS concept is a widely used method in the training design and competition planning due to its validity to reflect the maximal metabolic steady state (i.e., CP or CS) and the durability of the athletes above it (i.e., W' [work] or D' [distance]) (Hill, 1993). Its testing procedure requires the execution of two to five maximal efforts at the severe intensity domain, for which different models would fit the best regression line to the relationship between power or speed and the duration of the predicting trials (Hill, 1993). Accordingly, the accuracy of the model would be determined by the proximity of the predicting trials to the regression line, characterizing the derived parameters by a given standard error of the estimate (SEE). Larger SEE than 5% and 10% for CP/CS and W'/D' have been considered inaccurate models (Muniz‐Pumares et al., 2019). On the other hand, practitioners also extrapolate the regression line of the power‐ or speed‐duration curve applied to the so‐called non‐asymptotic models (i.e., the power or speed would decrease toward 0 with the increase of time) to estimate the performance at distances not yet attempted by the athletes (Ruiz‐Alias et al., 2024a). Thus, it raises the question of whether monitoring the speed or power output could influence the accuracy of these estimates.

In this context, it is noteworthy that the power output reported by the Stryd power meter has been considered a reliable metric, reporting a high interunit (coefficient of variation [CV] of ∼1.1%), interlimb (CV of ∼1.5%), and between‐day (CV of ∼1.6%) reliability in maximal efforts (Ruiz‐Alias et al., 2024b). Similarly, the spatiotemporal parameters reported by the Stryd IMU determining running speed (i.e., cadence and step length) have shown a high level of agreement with respect to gold standards (García‐Pinillos et al., 2021; Imbach et al., 2020), which provides the opportunity to record distance in contexts where GNSS is not feasible (i.e., treadmill and indoor track). On the other hand, it is well‐known that the distance captured by the GNSS signal of current sport watches is susceptible to different sources of error (i.e., satellite signal obstruction, satellite availability, weather conditions, and gaps in the data) (Scott et al., 2016), which could condition the proximity of the predicting trials to the best‐fit regression line.

According to the existing knowledge gaps, this study aims to compare the accuracy of the power output, measured by a power meter, with respect to the speed, measured by the Stryd IMU and a Garmin GNSS sport watch to determine the CP, CS, W', D', and long‐duration performance (i.e., 60 min).

## 2 MATERIALS AND METHODS

### 2.1 Experimental design

This study is part of a larger project investigating the modelling of running endurance performance through the power metric. The results related to the theoretical models and the selected time trials here used have been published elsewhere (Ruiz‐Alias et al., 2022, 2023a, 2023b, 2023c), while in this study, we compare the accuracy of the power metric with respect to the speed reported by the Stryd IMU and a Garmin GNSS sport watch. In a 4‐week training period, athletes performed seven time trials (i.e., 1, 3, 4, 5, 10, 20, and 60 min) in a randomized order. Testing sessions were preceded by two light training days and performed under similar environmental conditions (Temperature: 18–23 ºC; humidity: 30%–60%; and wind: <10 km/h), footwear, athletic track, and time of the day (±1 h). Each trial was monitored through the Stryd power meter, Stryd IMU, and a GNSS sport watch to determine the CP/CS, W'/D', and 60 min performance.

### 2.2 Subjects

Fifteen highly trained athletes (eight males and seven females, age: 23 ± 5 years, height: 1.66 ± 0.06 m, body mass: 58 ± 8 kg, training volume: 110 ± 15 km per week, and 5 km season‐best: 15:29 ± 00:53) participated in the study (McKay et al., 2021). All athletes were informed about the research purpose and procedures of the study before signing a written informed consent form. The study protocol adhered to the tenets of the Declaration of Helsinki and ethical approval was obtained by the institutional review board (No. 2288/CEIH/2021).

### 2.3 Time trials

The time trials were performed on a 400 m athletic track in lane 1. Athletes began the testing sessions with a standardized warm‐up which consisted of 10 min of running at low‐to‐moderate intensity (i.e., intensity corresponding to easy long‐running sessions). After a series of dynamic mobility exercises, three high‐intensity short bouts with 2 min of rest were performed as a part of the specific warm‐up. Then, athletes began the time trial under the instruction of completing the longest distance possible. The pacing was self‐selected and lap time feedback was given every 400 m. To avoid any training effect, two time trials were randomly performed each week except for the 60 min time trial (i.e., five different configurations: 1–5, 4–20, 3–10, and 60 min).

### 2.4 Power monitoring

The Stryd power meter (Stryd Summit Power Meter) was used to determine the mean absolute power output (W) of each time trial. The body mass was measured with a weight scale (Seca 813; Seca Ltd, Hamburg, Germany) and updated daily in the power meter. This one was always attached to the laces of the right footwear.

### 2.5 Distance monitoring

**FIGURE 1:**
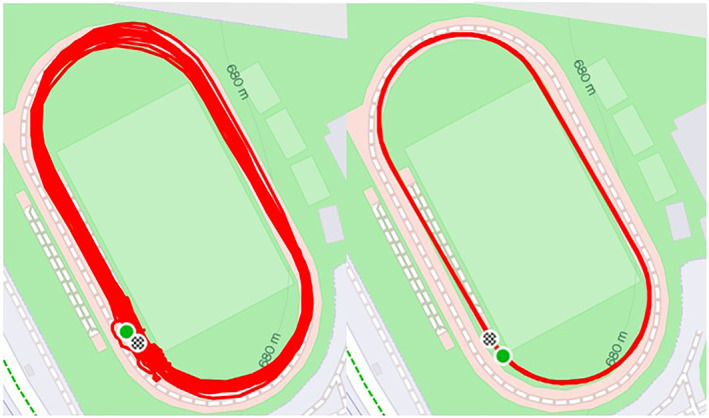
Examples of the GNSS sport watch outdoor and track running modes.

The Stryd IMU was also used to determine the distance covered in each time trial through the IMU's algorithms (Running speed = Cadence × Step length). Additionally, the distance covered was also monitored through the GNSS Garmin sport watch (Forerunner Music 245) using the Global Positioning System in combination with the GNSS (GLONASS). The time trials were recorded in “track run” mode, which utilizes specific algorithms designed to enhance the accuracy of the measures on a 400 m track (Garmin, 2024b). This mode is expected to provide more precise measurements compared to the “outdoor run” mode, where such algorithms are not applied (Figure 1).

### 2.6 CP/CS and W'/D'

Using the power and distance covered in the 3, 4, 5, 10, and 20 min trials, the CP/CS and W'/D' were defined as the interception and slope of the regression line between power or speed with the inverse of time (1/t, in seconds) (Ruiz‐Alias et al., 2023b; Whipp et al., 1982):

[*Power* = W´*(1/t) *+ CP*]

[*Speed* = D´*(1/t) *+ CS*]

### 2.7 Endurance performance estimation

Using the power and distance covered in the 1, 3, and 10 min trials (Ruiz‐Alias et al., 2023a), the 60 min performance was determined through the power law model (vii) (Kennelly, 1906):

Power*t = k*t^g^

Distance = k*t^g^

Where *k* is a constant that represents the maximal power or speed for one second and *g* is an exponent that indicates the decay of power or speed with the increase of time.

### 2.8 Statistical analysis

Descriptive data are presented as mean ± standard deviation (SD) and 95% confidence intervals. The coefficient of determination (*R* ^2^) of the CP and CS models was determined. The SEE of the CP/CS and W'/D' was calculated to compare the accuracy of the power and speed metrics. The CV ([SD/mean] *100]) between the actual and estimated 60 min performance reported through the power and speed metrics was also determined. Levene’s test was used to assess the homogeneity of variances of the SEE of CP/CS, W'/D', and 60 min performance. Lastly, a paired sample *t*‐test was performed to compare the actual and estimated 60 min performance when using the power and speed metrics. All statistical analyses were performed using the software package SPSS (IBM SPSS version 25.0) and statistical significance was set at an alpha level of 0.05.

## 3 RESULTS

### 3.1 CP/CS and W'/D'

Levene’s test revealed an unequal CP and CS variance (F_(2,42)_ = 79.7 and *p* < 0.001) (Table 1). Specifically, the SEE was 2.7% (2.1–3.3) when using the power metric, increasing to 3.4% (2.5–4.3) when monitoring speed with both the Stryd IMU and Garmin GNSS sport watch.

**TABLE 1:** Standard error of the CP/CS and W'/D' estimates when using the power and speed metrics.

	*R* ^2^	CP/CS (W or km/h)	SEE (W or km/h)	SEE (%)	Levene’s test	W'/D' (J or m)	SEE (J or m)	SEE (%)	Levene’s test
Power meter	0.95 ± 0.03	265 ± 59	7.1 (5.2–8.9)	2.7 (2.1–3.3)	F_(2,42)_ = 79.7; *p* < 0.001	14,341 ± 1189	1998 (1473–2522)	13.8 (10.4–17.3)	F_(2,42)_ = 12.4; *p* < 0.001
GNSS sport watch	0.88 ± 0.07	16.3 ± 1.9	0.5 (0.4–0.7)	3.4 (2.5–4.3)		745 ± 221	155 (116–193)	20.7 (16.6–24.7)	
IMU	0.88 ± 0.07	16.2 ± 1.9	0.5 (0.4–0.7)	3.4 (2.5–4.3)		747 ± 223	155 (116–194)	20.6 (16.7–24.7)	

*Note*: Mean ± standard deviation; (95% confidence interval); and *R* ^2^: coefficient of determination.

Abbreviations: CP, critical power; CS, critical speed; GNSS, global navigation satellite system; IMU, inertial measurement unit; SEE, standard error of the estimate; and W/D', work over CP or CS.

**FIGURE 2:**
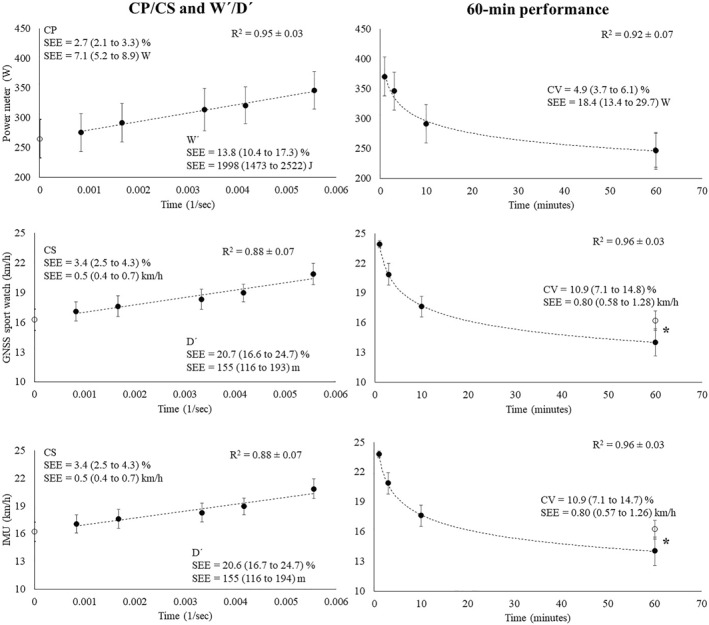
Intensity‐time modelling for the CP/CS, W'/D', and 60 min performance determination. CP, critical power, CS, critical speed, CV, coefficient of variation, W/D', work over CP or CS, and SEE: standard error of the estimate. *: Significant differences (*p* < 0.001); black and white dots illustrate the predicting and estimated values, respectively.

Levene’s test revealed an unequal W' and D' variance (F_(2,42)_ = 12.4 and *p* < 0.001). Specifically, the SEE was 13.8% (10.4–17.3) when using the power metric, increasing to 20.6% (16.7–24.7) when monitoring speed with both the Stryd IMU and Garmin GNSS sport watch (Figure 2).

### 3.2 Endurance performance estimation

Levene’s test revealed an unequal variance in the estimation bias of 60 min performance (F_(2,42)_ = 46.9 and *p* < 0.001) (Table 2). Specifically, the CV was 4.9% (3.7–6.1) when using the power metric, increasing to 10.9% (7.1–14.8) when monitoring speed with both the Stryd IMU and Garmin GNSS sport watch, resulting in nonsignificant differences between the actual and estimated 60 min power output (*p* = 0.853) and significant differences for the actual and estimated 60 min speed (*p* ≤ 0.001).

**TABLE 2:** Standard error of the 60 min performance estimate when using the power and speed metrics.

	*R* ^2^	Actual	Estimated	*p*‐value	SEE	CV (%)	Levene’s test
Power meter (W)	0.92 ± 0.07	247 ± 51	247 ± 56	0.853	18.4 (13.4–29.7)	4.9 (3.7–6.1)	F_(2,42)_ = 46.9; *p* < 0.001
GNSS sport watch (km/h)	0.96 ± 0.03	16.2 ± 1.7	14.0 ± 2.5	<0.001	0.80 (0.58–1.28)	10.9 (7.1–14.8)	
IMU (km/h)	0.96 ± 0.03	16.2 ± 1.7	14.0 ± 2.5	<0.001	0.80 (0.57–1.26)	10.9 (7.0–14.7)	

*Note*: Mean ± standard deviation; (95% confidence interval); and *R* ^2^: coefficient of determination.

Abbreviations: CV, coefficient of variation; GNSS, global navigation satellite system; IMU, inertial measurement unit; and SEE, standard error of the estimate.

## 4 DISCUSSION

This study aimed to compare the accuracy of the power output, measured by a power meter, with respect to the speed, measured by the Stryd IMU and a Garmin GNSS sport watch to determine the CP, CS, W', D', and long‐duration performance (i.e., 60 min). The results revealed that the power meter offered lower errors in the estimates than the speed reported by the Stryd IMU and Garmin GNSS sport watch, being therefore a more accurate metric for the athletes' monitoring on track.

The use of the power metric for modelling endurance running performance has gained solid acceptance. With regards to CP, Ruiz‐Alias et al. (2022) confirmed the validity of a simplified linear CP model of two time trials (i.e., 9 min and 3 min) for determining the power output associated with the second ventilatory threshold in highly trained athletes. Similarly, the stability of the CP when using two two time trials with respect to multiple points has also reinforced the utility of the power metric (Ruiz‐Alias et al., 2023c). Concerning the 60 min performance, Ruiz‐Alias et al. (2023a) confirmed the validity of different empirical models to estimate 60 min power output from shorter time trials (e.g., 1, 3, and 10 min). Pertaining W', although the power metric seems to improve the accuracy of this estimate with respect to the speed metric, this one is still characterized by a large SEE. Thus, its applicability to define the athletes' performance in the severe intensity domain has been questioned (Ruiz‐Alias et al., 2024c).

The validity of the GNSS for tracking distance has been a common concern among athletes. In this regard, Taddia et al. (2021) explored the level of agreement of a commercial GNSS sports watch with a small and GNSS lightweight prototype system for monitoring the running performance of a professional athlete in the Cooper and Kosmin tests. The results revealed a substantial difference of 3.7%–5.7% between them. Similarly, Lluch (Lluch et al., 2020) determined the accuracy of the most common commercial GNSS sport devices used in different marathon races, resulting in a significant overestimation of the 42,195 m (∼100–∼1400 m). Interestingly, GNSS sport watches oriented to road use were more precise than those oriented to trail or smartphone apps. Of note, the accuracy of these devices was also conditioned to the ﬁnish time, and the error being higher as the race took longer to finish. With regards to the specificity of the GNSS sport watch to the sport discipline, decent improvements have been reached through the track mode (See Figure 1), although the summed distance errors of each time trial resulted in higher SEE with respect to the power metric. The implications that these results could have on training prescription or pacing are of paramount importance.

Various nuances of testing warrant further discussion. On the one hand, it should be noted that although the power metric displayed a superior accuracy than the speed metric, both failed to replicate treadmill and outdoor performance (Ruiz‐Alias et al., 2024d; Triska et al., 2017). Similarly, it should be noted that the track mode has been created for 400 m tracks, for which, loops with other geometries than the standard shape would not be valid (Garmin, 2024b). In addition, athletes should be aware that this mode is limited to lane 1. In these cases, the power and speed metrics reported by the Stryd IMU could be a proper alternative, making this combination necessary for contextualizing power output (e.g., 4.5 W/kg resulted in 3:00 min/km). To this end, Garmin offers several sport faces which Stryd has collaborated with, creating a specific face for its power meter and IMU (Garmin, 2024a).

## 5 CONCLUSIONS

Athletes and practitioners concerned with the accuracy of their wearable devices should note that the Stryd power meter outperforms the Garmin GNSS sport watch in modelling endurance performance on the track. In this regard, it is also important to highlight the significant improvements made through the novel GNSS track mode, though this one is limited to standard 400 m tracks. Considering the novelty of the power metric in running, athletes and practitioners may find necessary its combination with running speed to put the power output metric in context. For this aim, similar performances were displayed by the Garmin GNSS and Stryd IMU. However, GNSS track mode limitations (i.e., just for running in 400 m tracks and in lane 1) should be considered when choosing between devices.

## CONFLICT OF INTEREST STATEMENT

The authors declare that they have no conflicts of interest.
